# High prevalence of *Clostridiodes diffiicle* PCR ribotypes 001 and 126 in Iran

**DOI:** 10.1038/s41598-020-61604-z

**Published:** 2020-03-13

**Authors:** Akram Baghani, Alireza Mesdaghinia, Ed. J. Kuijper, Amir Aliramezani, Malihe Talebi, Masoumeh Douraghi

**Affiliations:** 1 Division of Microbiology, Department of Pathobiology, School of Public Health, Tehran University of Medical Sciences, Tehran, Iran; 2 Center for Water Quality Research (CWQR), Institute for Environmental Research (IER), Tehran University of Medical Sciences, Tehran, Iran; 3 Department of Environmental Health Engineering, Faculty of Public Health, Tehran University of Medical Sciences, Tehran, Iran; 4 Department of Medical Microbiology, Center for Infectious Diseases, Leiden University Medical Center, Leiden, The Netherlands; 5 Department of Microbiology, School of Medicine, Iran University of Medical Sciences, Tehran, Iran; 6 Food Microbiology Research Center, Tehran University of Medical Sciences, Tehran, Iran

**Keywords:** Bacterial genetics, Epidemiology

## Abstract

*Clostridium difficile* is a leading causative agent of hospital-acquired and community-acquired diarrhea in human. This study aims to characterize the predominant *C. difficile* strains, RT001 and 126, circulating in Iranian hospitals in relation to resistant phenotypes, the antibiotic resistance genes, and their genetic relatedness. A total number of 735 faecal specimens were collected from patients suspected of CDI in Tehran hospitals. Typing and subtyping of the strains were performed using CE-PCR ribotyping and MLVA, respectively, followed by PCR assays for ARGs and indicators of Tns. Minimum inhibitory concentrations (MICs) of five antibiotics were determined by MIC Test Strips. Among 65 strains recovered from CDI patients, RT001 (32.3%) and RT126 (9.2%) were found as the most frequent ribotypes, and 64 MLVA types were identified. Using MLVA, RT001 and RT126 were subtyped into 6 and 4 groups, respectively. The *vanA*, *nim*, *tetM*, *gyrA*, *gyrB* genes were detected in 24.6%, 0%, 89.2%, 95.3%, and 92.3% of the strains, respectively. The indicators of Tns including *vanHAX*, *tndX*, and *int* were found in 0%, 3% and 29.2% of the strains, respectively. The most common amino acid (AA) alterations of GyrA and GyrB were related to substitutions of Thr82 → Val and Ser366 → Val, respectively. Resistance rate to metronidazole, vancomycin, tetracycline, ciprofloxacin, and moxifloxacin was 81.5%, 30.7%, 85%, 79%, and 74%, respectively. This study, for the first time revealed the subtypes of circulating RT001 and RT126 in Iran. It is of importance that the majority of the strains belonging to RT001 were multidrug resistant (MDR). This study also pointed to the intra-hospital dissemination of the strains belonging to RT001 and RT126 for short and long periods, respectively, using MLVA. The most important resistance phenotypes observed in this study was vancomycin-resistant phenotypes. Resistance to metronidazole was also high and highlights the need to determine its resistance mechanisms in the future studies.

## Introduction

*Clostridium difficile* is a leading causative agent of hospital-acquired (HA) and community-acquired (CA) diarrhea in human^1^. Severe *C. difficile* infections (CDIs) are commonly associated with hypervirulent strains pertaining to PCR ribotypes (RTs) 027 and 078. The *C. difficile* RT001 is regarded to be a hypervirulent strain as well as RT078^2^. *C. difficile* RT126 is considered as a hypervirulent strain that is genetically close to RT078 (identical patterns of PCR ribotyping)^3,4^. The RT078 is one of the most common types of *C. difficile* isolated from human in Europe^2,3^. The RT001 and RT072 associated with lethal CDI, are common in Europe^5,6^. The RTs001/072 have almost identical patterns of PCR ribotyping and could be differentiated by multilocus variable-number tandem repeat analysis (MLVA)^7^. Capillary electrophoresis (CE)-PCR ribotyping is currently employed as an appropriate alternative to agarose gel-based PCR ribotyping for epidemiological surveillance. However, it needs to be combined with MLVA in order to subtype the strains with identical patterns, and determine the genetic relatedness of the strains^3,8^.

Oral vancomycin and metronidazole are the most commonly used antibiotics for treatment of CDI^2,9^. Reduced susceptibility to vancomycin *C. difficile* strains has been increased within the recent decade^10^. Mechanisms of resistance to metronidazole have also remained elusive in *C. difficile*^10^. Several genes including *vanA*^11^, *rpoC*^12^, and *murG*^12^ are proposed to play a role in vancomycin resistance in *C. difficile*; however, resistance to vancomycin due to *vanA* could be of major concern as its horizontal transfer is likely to occur by Tn*1546*^13^. The high level of resistance to fluoroquinolones (mainly associated with amino acid substitutions in GyrA and/or GyrB^10^) has also been demonstrated in hypervirulent strains^14^. Tetracyclines are less likely to be linked to the induction of CDI; however, the tetracycline-resistant strains of *C. difficile* are clinically important. These strains may carry the tetracycline resistance genes (such as *tetM* and *tetW* genes) on the mobile genetic elements such as transposons (Tn) (i.e., Tn*916*, Tn*5397*, Tn*6190*, and Tn*6235*) possessing the ability of transferring genes inter- and intra-species^10,15^.

This study aims to characterize the predominant *C. difficile* hypervirulent strains, RT001 and 126, circulating in Iranian hospitals in relation to resistant phenotypes, the antibiotic resistance genes, and their genetic relatedness.

## Results

### 2-1- *C. difficile* strains

Out of 735 faecal specimens from cases suspected of CDI, 65 (8.8%) were positive for *C. difficile* by culture, L-prolin aminopeptidase test, and PCR for 16S *rRNA* gene. Of 65 strains, 8 (12.3%) were *tcdA* and *tcdB* negative and 57 strains (87.7%) were positive.

### 2-2- Antimicrobial susceptibility

Minimum inhibitory concentration (MIC) ranges were 0.125-256 mg/L, 0.032–256 mg/L, 0.023–256 mg/L, 0.047–32 mg/L, and 0.023–256 mg/L for vancomycin, metronidazole, tetracycline, ciprofloxacin, and moxifloxacin, respectively. Thirty percent (n = 20) of the strains were resistant to vancomycin. Resistance to metronidazole, tetracycline, ciprofloxacin, and moxifloxacin was 81.5% (n = 53), 84.6% (n = 55), 78.4% (n = 51), and 73.8% (n = 48), respectively. MIC50 and 90 were the same for metronidazole (≥256 mg/L), ciprofloxacin (≥32 mg/L), and moxifloxacin (≥32 mg/L) against strains belonging to RT001. In RT001, MIC50 and 90 of vancomycin were 1.5 and ≥256 mg/L and MIC50 and 90 of tetracycline were 32 and ≥256 mg/L. In RT126 strains, the MIC50 and 90 were as shown in Table 1.

**Table 1:** The MIC50 and 90 of antibiotics tested in *C. difficile* belonging to ribotypes 126.

Antimicrobial agent	MIC (mg/L)		
	Range	50	90
**Vancomycin**	0.5–2	0.875	2
**Metronidazole**	0.19–≥256	0.625	≥256
**Ciprofloxacin**	0.25–≥32	≥32	≥32
**Moxifloxacin**	0.5–≥32	16.5	≥32
**Tetracycline**	1.5–≥32	4	≥32

### 2-3- Antibiotic resistance genes

PCR assay for *vanA* was positive in 81.2% (13/16) of the vancomycin- resistant strains examined (Supplementary Table File). Nucleotide variation in *vanA* gene was Cytosine680 → Thymine (C680 → T). *vanHAX* as marker of Tn*1546* was not detected among the strains; therefore, absence of Tn*1546* was confirmed. No strain was positive for *nim* gene. The *gyrA* gene was detected in 95% of strains using the primer set gyrA1- gyrA2. Of them, 32.2% and 4.8% were found with amino acid (AA) substitution of Thr82 → Val and Thr82 → Ile, respectively. Using the gyrB1- gyrB2 primers, *gyrB* gene was detected in 92% of strains which possessed one of the following AA substitutions: Ser366 → Val (6.6%), Ser416 → Ala (6.6%), Ser366 → Ala (6.6%), Asp426 → Asn (3.3%), and Asp426 → Val (1.6%). PCR assay for *tetM* was positive in 86.2% (50/58) of the tetracycline- resistant strains. Twenty-seven and six percent and 3% of the strains contained the *int* and *tndX* genes as markers of Tn*916*-like and Tn*5397*-like, respectively. The presence of presumed antibiotic resistance genes in relation to the RTs is shown in Table 2. Odds ratio (OR) and *P* value were calculated for all resistance genes, and also mutation in *gyrA/B* genes in relation to resistance to the antibiotics. For some variables, OR was not applicable (NA), since at least one of the values was zero (Table 2).

**Table 2:** Presumed antibiotic resistance genes in relation to each PCR ribotype.

PCR ribotype	Presumed antibiotic resistance genes						
	*vanA*	*nimA*	*tetM*	*gryA*		*gryB*	
				Amino acid alterations			
	Number in susceptible *vs*. resistant						
**RT001**	0 *vs* 6^a^	0 *vs* 0	3*vs* 13^b^	**Ciprofloxacin**	**Moxifloxacin**	**Ciprofloxacin**	**Moxifloxacin**
				**Thr82Val:** 3*vs* 14^c^	**Thr82Val:** 1*vs* 16^d^	0 *vs* 0	0 *vs* 0
**RT126**	0 *vs* 0	0 *vs* 0	0 *vs* 6	**Thr82Ile:** 0 *vs* 1	**Thr82Ile:** 0 *vs* 1/6	**Ser366Val**/**Ser416Ala:** 1 *vs* 3 **Ser366Ala:** 0 *vs* 1	**Ser366Val**/**Ser416Ala:** 2 *vs* 2 **Ser366Ala:** 0 *vs* 1
**RT002**	0 *vs* 1	0 *vs* 0	0 *vs* 3	0 *vs* 0	0 *vs* 0	0 *vs* 0	0 *vs* 0
**RT003**	0	0 *vs* 0	1 *vs* 1	0 *vs* 0	0 *vs* 0	0 *vs* 0	0 *vs* 0
**RT005**	1 *vs* 0	0 *vs* 0	0 *vs* 1	0 *vs* 0	0 *vs* 0	0 *vs* 0	0 *vs* 0
**RT014**	1 *vs* 0	0 *vs* 0	1 *vs* 1	0 *vs* 0	0 *vs* 0	0 *vs* 0	0 *vs* 0
**RT023**	0 *vs* 0	0 *vs* 0	0 *vs* 1	0 *vs* 0	0 *vs* 0	0 *vs* 0	0 *vs* 0
**RT029**	0 *vs* 1	0 *vs* 0	0 *vs* 1	0 *vs* 0	0 *vs* 0	0 *vs* 0	0 *vs* 0
**RT037**	0 *vs* 0	0 *vs* 0	1 *vs* 0	0 *vs* 0	0 *vs* 0	0 *vs* 0	0 *vs* 0
**RT038**	0 *vs* 0	0 *vs* 0	0 *vs* 1	**Thr82Ile:** 0 *vs* 1	**Thr82Ile:** 0 *vs* 1	0 *vs* 0	0 *vs* 0
**RT039**	0 *vs* 0	0 *vs* 0	0 *vs* 1	0 *vs* 0	0 *vs* 0	0 *vs* 0	0 *vs* 0
**RT060**	0 *vs* 0	0 *vs* 0	0 *vs* 1	0 *vs* 0	0 *vs* 0	**Ser366Ala:** 0 *vs* 1	**Ser366Ala:** 0 *vs* 1
**RT072**	0 *vs* 2	0 *vs* 0	0 *vs* 2	**Thr82Val:** 1 *vs* 0	**Thr82Val:** 1 *vs* 0	0 *vs* 0	0 *vs* 0
**RT084**	0 *vs* 0	0 *vs* 0	0 *vs* 1	**Thr82Ile:** 0 *vs* 1	**Thr82Ile:** 0 *vs* 1	0 *vs* 0	0 *vs* 0
**RT097**	0 *vs* 0	0 *vs* 0	0 *vs* 1	0 *vs* 0	0 *vs* 0	0 *vs* 0	0 *vs* 0
**RT131**	0 *vs* 1	0 *vs* 0	0 *vs* 1	0 *vs* 0	0 *vs* 0	0 *vs* 0	0 *vs* 0
**RT266**	0 *vs* 0	0 *vs* 0	1 *vs* 1	0 *vs* 0	0 *vs* 0	0 *vs* 0	0 *vs* 0
**RT282**	0 *vs* 1	0 *vs* 0	0 *vs* 1	0 *vs* 0	0 *vs* 0	0 *vs* 0	0 *vs* 0
**RT389**	0 *vs* 0	0 *vs* 0	0 *vs* 1	0 *vs* 0	0 *vs* 0	**Ser366Ala**/**Asp426Val:** 0 *vs* 1	**Ser366Ala**/**Asp426Val:** 1 *vs* 0
**RT688**	1 *vs* 0	0 *vs* 0	0 *vs* 2	0 *vs* 0	0 *vs* 0	0 *vs* 0	0 *vs* 0
**RT700**	0 *vs* 0	0 *vs* 0	0 *vs* 1	0 *vs* 0	0 *vs* 0	0 *vs* 0	0 *vs* 0
**RT139**	0 *vs* 0	0 *vs* 0	0 *vs* 1	0 *vs* 0	0 *vs* 0	0 *vs* 0	0 *vs* 0
**Total**	3 *vs* 12	0 *vs* 0	7*vs* 42	4 *vs* 17	2 *vs* 19	1 *vs* 6	3 *vs* 4

^a^*P* value = 0.000.

^b^Odds Ratio (OR) with 95% confidence interval = 1.0, *P* value = 0.950.

^c^Odds Ratio (OR) with 95% confidence interval = 2.5, *P* value = 0.496.

^d^Odds Ratio (OR) with 95% confidence interval = 8.5, *P* value = 0.129.

The nucleotide sequences of *vanA* gene, *gyrA* and *gyrB* were deposited in GenBank under the accession numbers as indicated in Supplementary Table File.

### 2-4- PCR ribotypes

**Figure 1:**
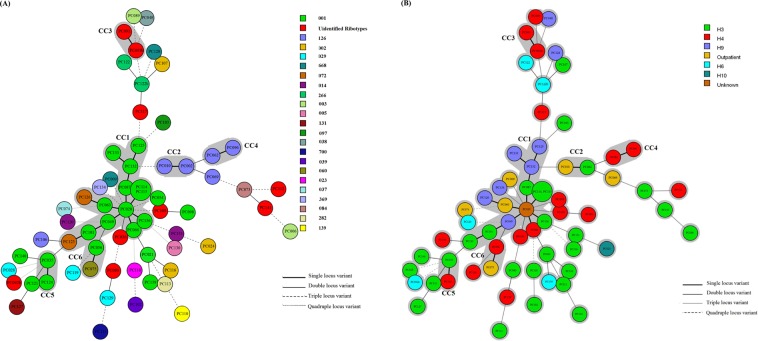
Minimum spanning tree representation (MST) based on Multiple-Locus Variable Number Tandem Repeat Analysis (MLVA) for *C. difficile* strains. The numbers in the circles represent code of strain. If more than one number is present in one circle, it represents the strains with 100% similarity in five variable-number tandem repeat (VNTR) loci. The CCs represent six clonal complexes with a single locus variant. (**A**) The colored circles indicate ribotypes (RTs) of *C. difficile* strains. (**B**) The colored circles indicate the isolation sources of *C. difficile* strains.

Of the 65 strains examined, 56 strains were assigned to 22 known RTs including RT001 (32.3%), RT002 (4.6%), RT003 (3.1%), RT005 (1.5%), RT014 (3.1%), RT023 (1.5%), RT029 (4.6%), RT037 (1.5%), RT038 (1.5%), RT039 (1.5%), RT060 (1.5%), RT072 (3.1%), RT084 (1.5%), RT097 (1.5%), RT126 (9.2%), RT131 (1.5%), RT266 (3.1%), RT282 (1.5%), RT369 (1.5%), RT688 (3.1%), RT700 (1.5%), and RT139 (1.5%). The remaining 9 strains (6.6%) not found in two database (*Clostridium difficile* Network for England and Northern Ireland (CDRN) and National Reference Laboratory for *Clostridium difficile* at University Medical Centre, Leiden) were designated as “unidentified RTs”. Two of 9 strains had the same chromatograms files (PC091 and PC091b). The chromatograms files of the unidentified RTs are presented in Supplementary Figure File. RT for each strain is indicated in Supplementary Table File. The non- toxigenic strains (PC048, PC073, PC075, PC080, PC091, PC091b, PC102, and PC141) belonged to RT038, RT084, RT060, RT102, and unidentified RTs (Supplementary Table File). The strains of RT001 were isolated from patients in different hospitals including H3, H4, H9 and H6. In terms of ward of admission in each hospital for strains belonging to RT001, the following are noted: (i) two strains (PC114 and PC115) from H3 were found in different wards “Nose Ear Trachea (NET) and Intensive Care Unit (ICU)” with two months interval (November and December 2016); (ii) two strains (PC054 and PC056) from H4 were isolated in the same month (on August 2015), albeit from different wards including “respiratory disease” and “nephrology”; (iii) two strains (PC132 and PC133) from H9 were isolated from different wards including “infectious disease” and “surgery 2” with two months interval (May and June 2017) (Fig. 1b, Supplementary Table File). Regarding RT126, three strains (PC062, PC096, and PC106) were isolated from patients admitted to the ward “medical 2” in hospital H4 within different months (Fig. 1b, Supplementary Table File).

### 2-5- MLVA types

Using MLVA, subtyping of the 65*C. difficile* strains with different RTs patterns revealed 64 MLVA types (Supplementary Table File) and 6 clonal complexes (CC1, CC2, CC3, CC4, CC5, and CC6) for RTs 001, 072, 126, 060, and unidentified RTs. Each of four CCs; for example, CC2 (RT126), CC3 (unidentified RT), CC4 (RT126), and CC5 (RT001) were made up of a single RT; while two other CCs including CC1 and CC6 contained different RTs. In other words, genetic relatedness (defined as single locus variant) was observed between RTs 001 and 072 in CC1, and also between RTs 001 and 60 in CC6. The CC1 as the largest CC included twelve RT001 and two RT072. In addition, two strains (PC114 and PC115) had identical MLVA types in CC1 (MLVA type 12) in all five loci (Supplementary Table File). The CC2 and CC4 included four out of six strains with RT126 pattern (Fig. 1a), while the CC2 possessed two isolates (PC002 and PC010) from H3 hospital and outpatient in August and September 2014 (Fig. 1b, Supplementary Table File). The CC3 contained two isolates (PC091 and PC091b) with the same RT pattern (unidentified RT) from the same ward (“respiratory disease”) of H4 hospital. Also, the CC4 included two isolates (PC062 and PC096) from the “medical 2” ward of H4 hospital during three months (from August to October 2016) (Fig. 1b, Supplementary Table File).

The CDR4, CDR5, and CDR9 did not generate any amplicon in 6 isolates with RT126 and one isolate with RT668) and were considered to be absent or null (Supplementary Table File). Locus CDR4 with DI = 0.945 was found to be the most variable among the loci, whereas the locus CDR48 with DI = 0.651 had the lowest variability for this population of *C. difficile* strains. DIs of the CDR9, CDR5, and CDR49 were 0.841, 0.872, and 0.935, respectively.

## Discussion

The CDI is a major medical threat to human health around the world. Most of the CDI cases reported recently in Europe are often associated with the hypervirulent strains such as RT027 and RT078^7^. Also, some of the *C. difficile* RTs such as RT001 are more likely to cause the severe CDI^1^. Accurate typing of *C. difficile* strains is crucial for defining whether a strain is sporadic or part of an outbreak. A study from Tehran, conducted on the isolates collected in 2011, showed that RT001 and RT126 are the most predominant RTs among the isolates of *C. difficile*^16^. The findings of the current study are consistent with those of the previous study by Azimirad *et al*., revealing that RT126 and RT001 are the most predominant RTs among *C. difficile* isolates^16^. In the current study, the faecal specimens were from patients while Azimirad *et al*., (2017), examined stool samples from hospitalized patients and swabs from environment as well as medical devices. Moreover, samples in their study were collected during 2011, whereas samples in our study were collected from 2011 to 2017. Also, an important point is that the subtyping of the isolates of different ribotypes by MLVA and the identification of resistance genes and transposons were performed for the first time in Iran. None of them have been carried out in Azimirad et *al*., study. Finding other different RTs in metropolitan Tehran might be arising from the difference in sample sizes, time periods and/or target hospitals. The European studies emphasized that RT001 is one of the RTs that are frequently recovered from patients^7^. The association between RT001 and the severe CDI has been reported^5^, which may be related to high-density of spore production by *C. difficile* RT001^17^. In this research, the severity of CDI in patients has not been recorded; thus, assessment of such an association was not possible. Here, we applied the CE-PCR ribotyping as a robust method for typing and identifying the predominant RTs of *C. difficile* strains circulating in Tehran during 2011–2017. Afterwards, MLVA was performed to determine the genetic relatedness of the *C. difficile* RTs. Moreover, the antibiotic-resistant phenotypes of the predominant *C. difficile* RTs and their antibiotic resistance genes were identified.

In this study, the CE-PCR ribotyping of *C. difficile* strains demonstrated the RT001 as the most prevalent RT, followed by RT126 as the second most prevalent RT among *C. difficile* strains from target hospitals. The RT001 and RT126 were distributed in different CCs based on MLVA. In the Middle East, the hypervirulent strains RT027, RT078, RT001 and also RT126 were not found as the most prevalent RT, while one of the most common RT in Qatar, Kuwait, and Lebanon were RT258, RT139, and RT014, respectively. RT078 has not been found in those studies, but RT027 has been found in 1.3% and 0.9% of isolates in Qatar and Lebanon, respectively^18–20^. Based on the observed allelic diversity, CDR4 with DI = 0.945 had the most discriminatory power of the loci tested; a finding that is consistent with a previous report by Marsh *et al*.^21^.

Interestingly, the majority of strains with RT001 pattern (90.4%) were resistant to metronidazole. Previous studies in Europe reported a reduced susceptibility to metronidazole in 2008 and 2012^22,23^. Of 21 strains belonging to RT001, 7 strains were resistant to vancomycin (MICs of 4 strains =≥ 256 mg/L, MIC of one strain = 4 mg/L, MIC of one strain = 6 mg/L). In this work, the majority of the strains belonging to RT001 were multidrug resistant (MDR), defined as resistance to at least 4 antibiotics including vancomycin, metronidazole, ciprofloxacin, moxifloxacin, and tetracycline. Resistance to fluoroquinolones in RT001 was also reported by Krutova *et al*., in Czech Republic^1^. As already reported, RT001 and RT072 are considered as one complex (RT001/072). Here, we noted that the RT001 and RT072 had close genetic relatedness and clustered in CC1; although, they were obtained from different hospitals (H3 and H9) (Fig. 1b). The genetic relatedness of RT001 and RT072 is also demonstrated previously in Europe^7^. More than half of the strains with RT001 pattern (57.1%) were located in CC1, even though they were obtained from the patients admitted to different hospitals (H3, H4, H9) or outpatients. The remaining strains belonging to RT001 were assigned to other CCs. Between November and December 2016, the strains belonging to RT001 including PC114 and PC115 were isolated from the same hospital (H3) in different wards. In August 2015, the strains PC054 and PC056 were isolated from the same hospital (H4) from different wards. Moreover, between May and June 2017, the strains PC132 and PC133 were isolated from the same hospital (H9) in different wards. The close genetic relatedness of the strains including PC114, PC115, PC132, and PC133 was also observed and confirmed by MLVA method (located in CC1 with difference in one locus). It is necessary to mention that RT001 was subtyped in different CCs, therefore; MLVA could subtype the identical RTs as it has already been found by Krehelova *et al*., in Slovakia^24^. It is important to remind that PC114 and PC115 had an identical MLVA type and were assigned in CC1; one may conclude that both strains have probably had a common ancestor. Antibiotic resistance pattern was similar in both strains except for fluoroquinolones. Although both strains were resistant to tetracycline, but with different MICs (PC114: MIC = 12 mg/L, PC115: MIC ≥ 256 mg/L), *tetM* gene was only found in one strain. It seems that different mechanisms might be implicated in the resistance to tetracycline in both strains.

In the current study, the RT126 was found as the second most prevalent RT among the *C. difficile* strains examined. All strains with RT126 pattern were susceptible to vancomycin and resistant to tetracycline. However, five of the six strains with RT126 pattern were susceptible to metronidazole and three of the six strains were susceptible to fluoroquinolones. While the strains with RT126 were detected, *C. difficile* RT078 was not observed among the strains. The RT078 and RT126 or RT078-like are considered as hypervirulent strains. They possess the 39 bp deletion in *tcdC* gene and produce binary toxin. They have similarity in banding patterns in agarose gel-based PCR ribotyping method and can be differentiated with the CE-PCR ribotyping^25^. Presence and detection of RT126 is important, because the severe CDI cases are associated with RT126 or RT078-like clone^26^. In France, RT078/126 was the second most prevalent RT resistant to tetracycline^27^ similar to finding of the present study. It is noteworthy that CC4 contained two *C. difficile* RT126 strains (PC062 and PC096) isolated from the same ward (Medical 2) of hospital H4 with close genetic relatedness. Antibiotic resistance pattern was similar in both strains except for metronidazole. Considering the date of isolation for both strains (November 2015 and August 2017), it is concluded that these strains have had high durability in the ward of a single hospital. With regard to RT014, it was only accounted for 3% of strains in this study, while found as one of the most common RTs in European countries^7^.

In the present research, resistance to vancomycin was detected (4 strains; MIC = 4–16 mg/L and 16 strains; MIC ≥ 256 mg/L). Vancomycin reduced susceptibility has been observed in the last years (MICs ≥ 4 mg/L)^10,28^. Recently, the intermediate-resistance to vancomycin (MIC = 4 mg/L) has been noted in the isolates from various regions of Europe^10^. Resistance to vancomycin (MIC ≥ 8 mg/L) was previously observed in other studies among the isolates belonging to various RTs including RT027, RT126, RT001, RT072 and RT356^8,10^. The presence of *vanA* gene is associated with the resistance to vancomycin in strains of RT001 and reach a statistical significance (*P* = 0.001). However, none of the strains with RT126 pattern had *vanA* gene and were resistant to vancomycin (Table 2). Noren *et al*., in Sweden reported three isolates belonging to RT002 with vancomycin MICs = 4–8 mg/L in a patient who received vancomycin for treatment of septicaemia of *Staphylococcus aureus*^29^. In 1988, vancomycin-resistant *Enterococcus faecium* and *Enterococcus faecalis* containing *vanA* resistance locus were isolated and reported in England for the first time^13^. In the current study, PCR sequencing of *vanA* gene confirmed the presence of this gene in 24.2% of the strains (3 vancomycin susceptible strains and 13 vancomycin resistant strains). Presence of *vanA* gene may play a role in the resistance to vancomycin in strains examined here, because *vanA* gene was detected in most of the vancomycin-resistant strains (13/16 strains or 81.2%), even though the presence of Tn*1546* was not confirmed by *vanHAX* amplification. It is possible that the expression of *vanA* gene does not always occur in the strains; therefore; that is why the *vanA* gene was detected in vancomycin susceptible strains. Detection of *vanA* gene in *C. difficile* was performed by Barkin *et al*., and it was found in 6.1% of strains examined^11^.

The reduced susceptibility to metronidazole was observed in more than half of the strains (12 strains; MIC > 2 mg/L, 1 strain; MIC = 6 mg/L, and 52 strains; MIC ≥ 256 mg/L). A study in England reported reduced susceptibility to metronidazole in 24.4% of isolates with RT001 pattern^22^. In a study conducted by Husain *et al*., the *nimA-J* genes have not been detected in the metronidazole-resistant phenotypes^30^. Detection of *nimA* was performed by Barkin *et al*., and it was found in 47.5% of strains^11^. Since *nimA* gene was not observed in any strains examined here, probably other mechanisms may contribute to resistance to metronidazole. No *nimA* gene was also found in *C. difficile* isolates in a study conducted by Plaez *et al*.^31^. Recently, the pCD-METRO plasmid was detected in metronidazole resistant strains by Boekhoud *et al*., and suggested as the first metronidazole resistance mechanism that is mediated by plasmid^32^. The other resistance mechanisms; for examples, mutations within germination (*cspC*) loci, sporulation (*spo0A*), ferric uptake regulator (*fur*) and corporphyrinogrn III oxidase gene (*hemN*) could be linked to resistance to metronidazole^10^ and need to be tested in the future studies.

More than half of the strains (51/65 or 78%) were resistant to ciprofloxacin and 43 among 51 strains (43/51 or 84%) were also resistant to moxifloxacin (co-resistance), while 8 among 51 strains (8/51 or 16%) were susceptible to moxifloxacin Since the fluoroquinolones should be noted for their propensity to induce CDI^10^, highly-resistant strains were expected to be observed. The substitution Thr82 → Ile in GyrA has been reported as a predominant mechanism of resistance to fluoroquinolones particularly in RT027^33^. It is because the predominant substitution in the strains examined here was due to the substitution Thr82 → Val. The substitution Thr82 → Ile in GyrA was observed in the fluoroquinolones-resistant strains (PC010, PC048, and PC073) with different RTs patterns (RT126, RT038, and RT084) and different isolation sources (outpatients, H9, and H3). In the present work, the substitution Thr82 → Val in GyrA was not associated with the resistance to fluoroquinolones, since this substitution was found in susceptible strains as well. The substitutions Ser416 → Ala, Ser366 → Ala, and Asp426 → Asn in GyrB were detected in susceptible strains as well as resistant strains in this study, in agreement with the previous reported data from Europe^33,34^. Therefore; these substitutions do not appear to have a key role in the resistance to ciprofloxacin and moxifloxacin. It was previously reported that one of the moxifloxacin-resistant *C. difficile* isolates had the substitution Asp426 → Val in GyrB^33^, while in the present study it was observed in one moxifloxacin-susceptible strain (PC134) with RT369 pattern. In contrary to the findings by Huang *et al*.^34^, that had found the substitution Asp426 → Val in GyrB of *tcdA* negative/*tcdB* positive strains, this substitution was found in *tcdA* positive and *tcdB* positive strain (PC134) in the current study. In this research, it is noted that the mutation rate in GyrA was higher than GyrB. This finding was previously reported in 2009 by Huang *et al*.^34^. The mutations in *gyrA* gene were observed in most of fluoroquinolones- resistant strains, but this observation was not statistically significant (OR = 2.5, *P* value=0.496 and OR = 8.5, *P* = 0.129) (Table 2).

In the present study, most *C. difficile* strains examined were resistant to tetracycline. In RT001 strains, the *tetM* gene was found in most of the tetracycline- resistant strains, but this concordance was not statistically significant (OR = 1, *P* = 0.950) (Table 2). The *tetM gene* was observed in all strains RT126 which were resistant to tetracycline. The *tetM* gene is a predominant mechanism of resistance to tetracycline. Here, two strains with RT060 (PC075 strain with MIC = 0.38 mg/L) and RT039 (PC102 strain with MIC ≥ 32 mg/L) contained Tn*5397*-like. Interestingly, all of the strains with RT126 pattern were resistant to tetracycline and harbored Tn*916*-like (except one strain; PC062). In the tetracycline-resistant strains with no detection of Tn*5397*-like and Tn916-like, probably other mechanisms are involved in the emergence of the resistant phenotypes. Spigaglia *et al*., reported the RT012 and RT048 strains carrying *tetM* and *tetW* genes^35^. In a study by Bakker *et al*., Tn*916*-like was found in all tetracycline-resistant *C. difficile* strains^36^.

The hypervirulent strains RT027 and RT078 were not detected in Tehran, although they are more frequently found in Europe and USA. Due to lack of data regarding the RTs circulating in the Middle East, the genetic pattern of hypervirlent strains is not clearly evident in this geographical region as it is in Europe and USA. To reach a clear conclusion, several studies are needed to identify the widespread hypervirluent strains in this area. In accordance with the previous study in Tehran, the RT001 and RT126 were dominant. A notable point is that the majority of the strains belonging to RT001 were multidrug resistant (MDR). The rate of vancomycin resistant strains of *C. difficile* reached an alarming level. This study, for the first time revealed the subtypes of circulating RT001 and RT126 in Iran, and shed light on the genetic relatedness of the predominant RTs using MLVA. MLVA could subtype the same RTs in different CCs; RT001 in 4 groups as well as RT126 in 4 groups. This study also pointed to the intra-hospital dissemination of the strains belonging to RT001 and RT126 for short and long periods, respectively. The most important resistance phenotypes observed in our study was vancomycin-resistant phenotypes; however, there is a need to assess the role of other presumed genes.

## Materials and methods

### 4-1- Bacterial isolates

A total of 735 faecal specimens were obtained from hospitalized patients (inpatients) from 5 hospitals in Tehran including H3, H4, H6, H9, H10, and outpatients aged 2 to 95 years old. Other faecal specimens were obtained from patients whom their settings were not recorded. All of patients were suspected to CDI and had watery diarrhea and tested for *C. difficile* at anaerobic bacteriology laboratory (ABL) in Tehran, Iran.

After alcohol shock, faecal specimens were cultivated on cycloserine cefoxitin fructose agar (CCFA). For anaerobic conditions, the jars were attached to Anoxomat device with a gas mixture of 80% nitrogen, 10% hydrogen, and 10% carbon dioxide, and then incubated at 37 °C for 48 hours. Isolates were identified as *C. difficile* by colony morphology, the typical odor of *C. difficile*, and a positive test of L-prolin aminopeptidase. Total genomic DNA was extracted by Chelex® 100 (Sigma, USA) from fresh cultures grown on Brucella agar plates supplemented with 5% sheep blood. *C. difficile* strains were identified by PCR assay using the primer set described by Kikuchi *et al*., for 16S *rRNA* gene^37^. *C. difficile* strains were also screened for *tcdA* and *tcdB* genes^38,39^.

### 4-2- Antimicrobial susceptibility testing (AST)

*In vitro* susceptibility to vancomycin, metronidazole, tetracycline, ciprofloxacin, and moxifloxacin was determined by MIC Test Strips (Liofilchem, Italy) with gradient antibiotic range from 0.016 to 256 mg/L for vancomycin, metronidazole, and tetracycline and 0.002 to 32 mg/L for ciprofloxacin and moxifloxacin. The medium and the strain for quality control were brucella blood agar and *C. difficile* 630, respectively. The breakpoints 2, 2, 0.25, 4 and 4 mg/L were applied for vancomycin, metronidazole, tetracycline, ciprofloxacin, and moxifloxacin, respectively^23^.

### 4-3- Detection of antibiotic resistance genes and transposon markers

*C. difficile* strains were examined by PCR for the presence of the antibiotic resistance genes including *vanA*, *nim*, *tetM*, *gyrA*, and *gyrB*. The *vanA* and *nim* genes, with resistance to vancomycin and metronidazole, were detected by amplification of 1030 bp and 458 bp fragments, respectively using the specific primers as described previously^40,41^. Also, *vanHAX* fragment was amplified with the primer pairs vanH1-vanX2 described by Yu *et al*., for detection of Tn*1546*^42^. Using PCR, we examined the presence of *tetM* gene, responsible for resistance to tetracycline using primers TETMd-TETMr^43^. The isolates were examined for the presence of the *int* and *tndX* genes, which encode two proteins in the Tn*916*-like and Tn*5397*-like family of conjugative transposons, respectively using primer pairs INTf-INTr and tndX1- tndX3^44,45^. The genes including *gyrA* and *gyrB* genes were amplified with the primers gyrA1- gyrA2 and gyrB1- gyrB2, respectively^33^.

PCR products of *vanA*, *gyrA* and *gyrB* were sent to Macrogen, Inc. company (Seoul, Korea) and sequenced on an Applied Biosystems 3730xl DNA Analyzer (CA, USA) using either PCR primer. The sequences were compared with other sequences using the Basic Local Alignment Search Tool (BLAST) server of the National Center for Biotechnology Information (NCBI).

### 4-4- CE-PCR ribotyping

For CE-PCR ribotyping, amplification of the 16S-23S *rRNA* intergenic spacer region was conducted by PCR according to the protocol previously described^8^. *C. difficile* strain 630 (RT012) was used as a control. After sending the chromatogram files (.fsa format) to two laboratories including National Reference Laboratory for *Clostridium difficile* at University Medical Centre, Leiden and *Clostridium difficile* Ribotyping Network for England and Northern Ireland (CDRN), the RTs were assigned for each *C. difficile* strain.

### 4-5- Multiple-locus variable-number tandem-repeat analysis (MLVA) typing

The MLVA assay in the present study is based on capillary electrophoresis of the five most polymorphic loci. PCR amplification of the five selected *C. difficile* repeat (CDR) loci was performed in singleplex format in a 20 µl final volume containing 0.2 unit *Taq* DNA Polymerase 2x Master Mix (Ampliqon, Denmark) and 0.2 mM concentrations of each primer on *C. difficile* strains with the primer sets of 6-carboxyfluorescein (6Fam)-CDR4F-CDR4R, hexachlorofluorescein (Hex)-CDR9F-CDR9R, 6Fam-CDR5F-CDR5R, 6Fam-CDR48F-CDR48R and Hex-CDR49F-CDR49R that were published previously by Marsh *et al*.^21,46^.

PCR products were analyzed by an Applied Biosystems 3730xl DNA Analyzer (CA, USA) with a LIZ1200 marker as an internal marker for each sample. The *C. difficile* 630 strain was included in all MLVA PCR amplification runs and analyses to act as a control standard. Raw allele data were analyzed using Peak scanner (v1.0, Applied Biosystem). Minimum spanning tree (MST) was created using Bionumerics software v7.6 (Applied Maths, Belgium) with default settings including two priority rules (locus variants with a weight of 10000 and locus variants with weight of 10). When the number of repeats in four out of five loci (tolerance = 1) were the same, the strains were considered as clonal complex (CC). Copy numbers at each of the five CDR loci were concatenated to generate an MLVA type for each isolate (Supplementary Table File).

Diversity index was calculated for the MLVA loci; Diversity index (DI) = 1-Σ nj (nj-1)/N (N-1) where n = total number of alleles of a particular locus and N = the total number of alleles across all loci^47^.

### Ethical approval

The ethics committee of Tehran University of Medical Sciences approved the study. All patients have signed the informed consent for giving the faecal specimens for research. Parents/legal guardian provided written informed consent for their child under the age of 18 years to participate in this study. All laboratory procedures have been performed in accordance with the guidelines of EUCAST, CLSI, and published paper. The metronidazole and vancomycin resistant strains will be send for confirmation to the Reference Laboratoy at LUMC.

## Supplementary information

Supplementary Figure File.

Supplementary Table File.

## Data Availability

The authors declare that all data presented in this study are available within the article and its Supplementary Information Files or can be available from the corresponding author upon request. DNA sequences of the genes that have been deposited in GenBank are available in https://www.ncbi.nlm.nih.gov/.
