# Supplementary material for: High prevalence of Clostridiodes diffiicle PCR ribotypes 001 and 126 in Iran
Source: Sci Rep. 2020 Mar 13;10:4658. doi: 10.1038/s41598-020-61604-z (PMC7070088; doi:10.1038/s41598-020-61604-z)
Supplement: Supplementary file 1 — Supplementary Figure File. [file 41598_2020_61604_MOESM1_ESM.pdf]

High prevalence of *Clostridiodes diffiicle* PCR ribotypes 001 and 126 in Iran

Akram Baghani[1], Alireza Mesdaghinia[2,3], Ed J. Kuijper[4], Amir Aliramezani[1], Malihe Talebi[5], Masoumeh Douraghi*[1,6]

[1]Division of Microbiology, Department of Pathobiology, School of Public Health, Tehran University of Medical Sciences, Tehran, Iran.

[2]Center for Water Quality Research (CWQR), Institute for Environmental Research (IER), Tehran University of Medical Sciences, Tehran, Iran.

[3]Department of Environmental Health Engineering, Faculty of Public Health, Tehran University of Medical Sciences, Tehran, Iran

[4]Department of Medical Microbiology, Center for Infectious Diseases, Leiden University Medical Center, Leiden, the Netherlands.

[5]Department of Microbiology, School of Medicine, Iran University of Medical Sciences, Tehran, Iran.

[6]Food Microbiology Research Center, Tehran University of Medical Sciences, Tehran, Iran.

*** Corresponding author**

Masoumeh Douraghi, Ph.D., Division of Microbiology, Department of Pathobiology, School of Public Health, Tehran University of Medical Sciences, Tehran, Iran, PO Box: 14155-6446, Fax: +98 21 88954913 Tel: +98 21 42933152, Email: mdouraghi@tums.ac.ir

Unidentified ribotype of PC036

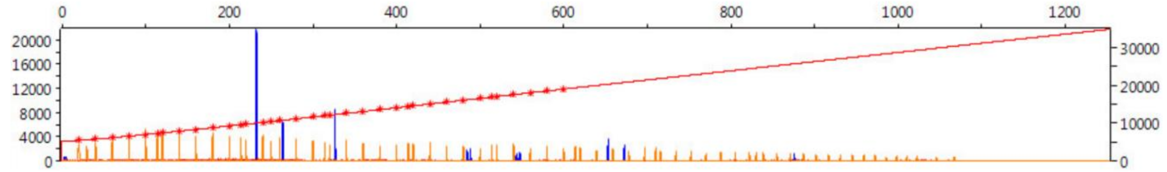

Unidentified ribotype of PC080

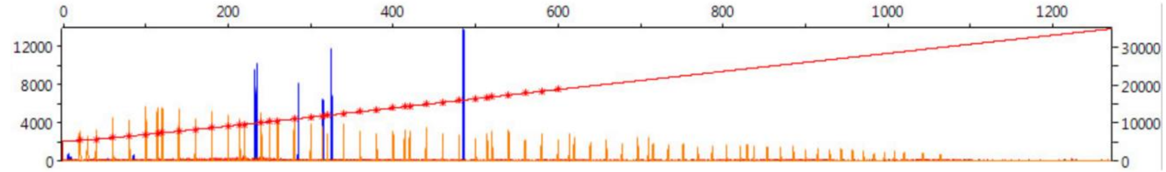

Unidentified ribotype of PC091 and PC091b

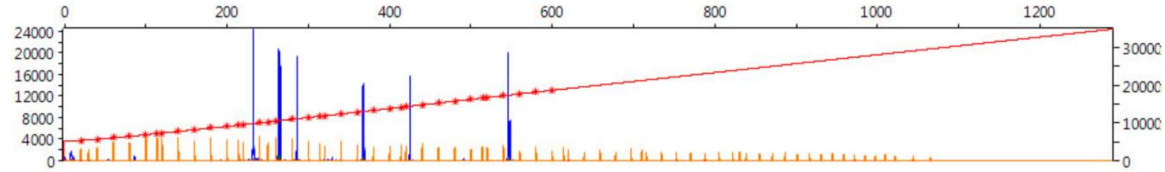

Unidentified ribotype of PC092b

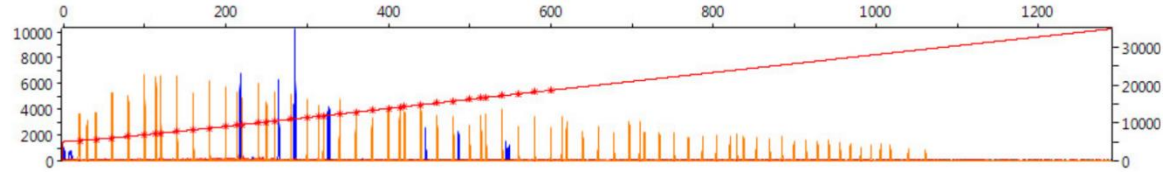

Unidentified ribotype of PC109

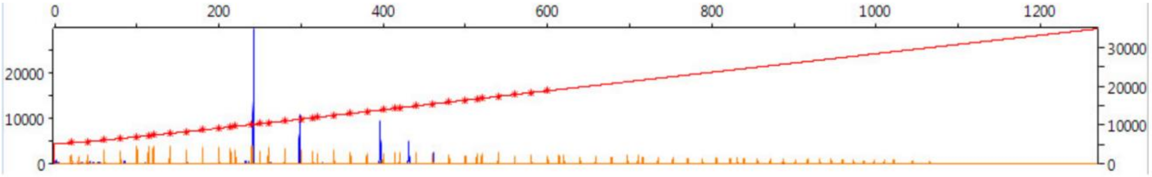

Unidentified ribotype of PC112

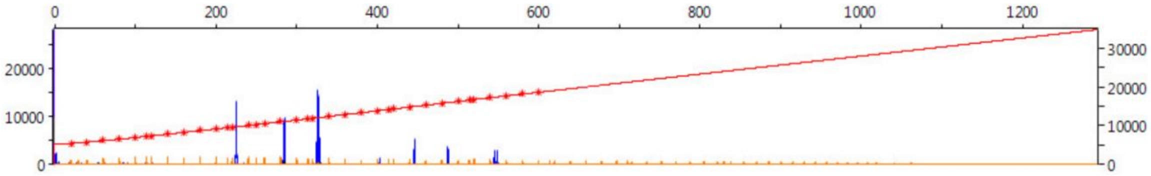

Unidentified ribotype of PC117

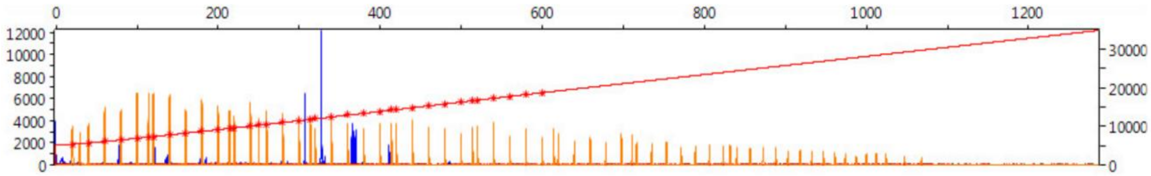

Unidentified ribotype of PC141

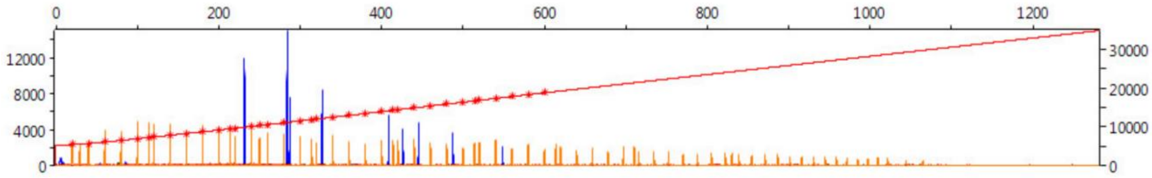

Supplementary figure file: The chromatogram files (.fsa) of the Unidentified ribotypes for 9 *C. difficile* strains. The vertical and horizontal axes show the peak-size (bp) and the peak-height (fluorescent units) of ribosomal bands, respectively.
