# Supplementary material for: High prevalence of Clostridiodes diffiicle PCR ribotypes 001 and 126 in Iran
Source: Sci Rep. 2020 Mar 13;10:4658. doi: 10.1038/s41598-020-61604-z (PMC7070088; doi:10.1038/s41598-020-61604-z)
Supplement: Supplementary file 2 — Supplementary Table File. [file 41598_2020_61604_MOESM2_ESM.pdf]

High prevalence of *Clostridiodes diffiicle* PCR ribotypes 001 and 126 in Iran

Akram Baghani[1], Alireza Mesdaghinia[2,3], Ed J. Kuijper[4], Amir Aliramezani[1], Malihe Talebi[5], Masoumeh Douraghi*[1,6]

[1]Division of Microbiology, Department of Pathobiology, School of Public Health, Tehran University of Medical Sciences, Tehran, Iran.

[2]Center for Water Quality Research (CWQR), Institute for Environmental Research (IER), Tehran University of Medical Sciences, Tehran, Iran.

[3]Department of Environmental Health Engineering, Faculty of Public Health, Tehran University of Medical Sciences, Tehran, Iran

[4]Department of Medical Microbiology, Center for Infectious Diseases, Leiden University Medical Center, Leiden, the Netherlands.

[5]Department of Microbiology, School of Medicine, Iran University of Medical Sciences, Tehran, Iran.

[6]Food Microbiology Research Center, Tehran University of Medical Sciences, Tehran, Iran.

*** Corresponding author**

Masoumeh Douraghi, Ph.D., Division of Microbiology, Department of Pathobiology, School of Public Health, Tehran University of Medical Sciences, Tehran, Iran, PO Box: 14155-6446, Fax: +98 21 88954913 Tel: +98 21 42933152, Email: mdouraghi@tums.ac.ir

Supplementary table file. The isolation source and genotypic characteristics of the 65 *C. difficile* strains examined in this study

Code	Data of sampling	Isolation source	Hospital Wards	RT	MLVA result (number of tandem repeats for each locus)					MLVA type	Nucleotide variation in *vanA*/ Accession number	*tetM/ int/tndX*	AA substitution in GyrA/ Accession number	AA substitution in GyrB/ Accession number
					CDR4	CDR9	CDR5	CDR48	CDR49					
PC002	2014 August	H3	NR[a]	RT126	0	12	0	8	25	1	-[b]	+/+/-	WT[c]/ MH769591	Ser366 → Val, Ser416 → Ala/ MH796019
PC006	2014 August	H3	NR	RT003	27	4	3	6	14	2	-	+/+/-	WT/ MH769630	WT/ MH796029
PC009	2014 August	Outpatient	Outpatient	RT668	8	0	14	8	7	3	-	+/-/-	WT/ MH769631	WT/ MH796030
PC010	2014 September	Outpatient	Outpatient	RT126	0	12	0	8	16	4	-	+/+/-	Thr82 → Ile/ MH769592	Ser366 → Ala/ MH796020
PC020	NR	NR	NR	RT001	32	5	14	8	13	31	-	-/-/-	Thr82 → Val/ MH769593	WT/ MH796031
PC021	2014 November	H3	Medical 2	RT001	34	6	14	8	10	62	-	-/-/-	-	-

PC024	2014 December	H10	Respiratory diseases	RT002	25	4	8	7	21	61	-	+/+/-	WT/ MH769594	WT/ MH796032
PC028	2015 February	H3	Liver transplantation	RT029	30	16	19	7	9	32	-	+/-/-	WT/ MH769632	-
PC035	2015 April	H3	General ICU	RT001	30	5	16	8	14	33	-	+/+/-	WT/ MH769595	WT/ MH796033
PC036	2015 April	H4	Internal	unidentified RT	30	5	14	6	13	63	-	-/-/-	WT/ MH769596	WT/ MH796034
PC048	2015 August	H9	NR	RT038	28	13	2	5	9	34	-	+/+/-	Thr82 → Ile/ MH769597	WT/ MH796035
PC049	2015 August	H9	Medical ICU	RT001	32	5	14	8	15	35	-	+/+/-	Thr82 → Val/ MH769598	WT/ MH796036
PC054	2015 August	H4	Respiratory diseases	RT001	38	5	9	8	20	36	-	+/-/-	Thr82 → Val/ MH769599	WT/ MH796037
PC056	2015 August	H4	Nephrology	RT001	30	5	2	8	15	37	-	-/-/-	Thr82 → Val/ MH769600	WT/ MH796038
PC062	2015 November	H4	Medical 2	RT126	0	0	0	8	28	38	-	+/-/-	WT/ MH769601	-
PC063	2015 November	Outpatient	Outpatient	RT001	35	5	14	8	13	39	-	-/-/-	Thr82 → Val/ MH769602	WT/ MH796039

PC066	2015 December	H4	Medical 2	RT001	34	5	14	8	13	40	-	+/+/-	Thr82 → Val/ MH769603	WT/ MH796040
PC069	2016 January	Outpatient	Outpatient	RT126	0	11	0	8	20	41	-	+/+/-	WT/ MH769604	Ser366 → Val, Ser416 → Ala/ MH796021
PC073	2011 August	H3	NR	RT084	32	11	3	5	20	42	-	+/+/-	Thr82 → Ile/ MH769605	WT/ MH796041
PC074	NR	NR	NR	RT037	35	6	4	3	21	43	-	+/-/-	WT/ MH769633	WT/ MH796042
PC075	NR	NR	NR	RT060	30	22	2	8	15	44	-	+/-/+	WT/ MH769606	Ser366 → Ala/ MH796022
PC080	2016 March	H3	NR	unidentified RT	50	12	2	6	13	48	-	+/+/-	WT/ MH769607	Ser366 → Ala/ MH796023
PC087	2016 June	H3	Infectious disease	RT001	27	5	14	8	13	5	-	+/-/-	Thr82 → Val/ MH769608	WT/ MH796043
PC089	2016 July	H4	Emergency unit	RT003	32	4	2	5	17	45	-	+/-/-	WT/ MH769634	Asp426 → Asn/ MH796024
PC091	2016 July	H4	Respiratory diseases	unidentified RT	19	9	2	5	16	57	-	+/-/-	WT/ MH769609	WT/ MH796044
PC091b	2016 August	H4	Respiratory diseases	unidentified RT	33	9	2	5	16	49	-	+/-/-	WT/ MH769635	WT/ MH796045

PC092b	2016 August	H6	CCU4	unidentified RT	31	9	5	2	14	51	-	+/-/-	WT/ MH769610	WT/ MH796046
PC096	2016 August	H4	Medical 2	RT126	0	0	0	8	14	46	-	+/+/-	WT/ MH769611	Ser366 → Val, Ser416 → Ala/ MH796025
PC098	2016 August	H4	Internal	RT001	23	10	15	8	19	47	-	+/-/-	WT/ MH769636	WT/ MH796047
PC101	2016 September	H3	Medical	RT001	23	5	14	8	15	6	-	+/-/-	Thr82 → Val/ MH769612	WT/ MH796048
PC102	2016 September	H3	Infectious diseases	RT039	25	1	3	2	11	7	-	+/-/+	WT/ MH769637	WT/ MH796049
PC103	2016 September	H3	Gastroenterolo gy	RT097	28	5	4	2	24	8	-	+/-/-	WT/ MH769638	WT/ MH796050
PC106	2016 October	H4	Medical 2	RT126	0	5	16	8	31	9	-	+/+/-	WT/ MH769613	Ser366 → Val, Ser416 → Ala/ MH796026
PC107	2016 October	H3	Infectious disease	RT002	22	9	7	8	32	10	-	+/-/-	WT/ MH769639	WT/ MH796051
PC109	2016 November	H4	Internal	unidentified RT	19	5	15	8	19	58	-	-/-/-	-	-
PC110	2016 November	H3	Surgery	RT139	12	15	6	8	11	59	-	+/-/-	WT/ MH769614	WT/ MH796052

PC111	2016 November	H3	Medical 2	RT700	34	10	2	6	9	52	-	+/-/-	WT/ MH769640	WT/ MH796053
PC112	2016 November	H4	Post HSCT	unidentified RT	32	7	7	6	20	60	-	+/-/-	-	-
PC113	2016 December	H3	Infectious disease	RT282	33	6	6	8	18	11	C680 → T/ MH66256	+/-/-	WT/ MH769641	WT/ MH796054
PC114	2016 November	H3	NET	RT001	32	5	14	8	4	12	WT/ MH666051	-/-/-	Thr82 → Val/ MH769615	WT/ MH796055
PC115	2016 December	H3	ICU	RT001	32	5	14	8	4	12	C680 → T/ MH753337	+/-/-	Thr82 → Val/ MH769616	WT/ MH796056
PC116	2017 January	H3	Liver transplantation	RT002	27	6	8	8	10	13	C680 → T/ MH753338	+/-/-	WT/ MH769642	WT/ MH796057
PC117	2017 January	H4	Hematology	unidentified RT	28	10	2	8	17	64	-	+/-/-	WT/ MH769617	WT/ MH796058
PC118	2017 January	H3	Hematology	RT023	34	7	3	2	16	14	-	+/-/-	WT/ MH769643	WT/ MH796059
PC119	2017 January	H4	Respiratory diseases	RT029	30	14	2	6	7	15	C680 → T/ MH753339	+/-/-	WT/ MH769644	WT/ MH796060
PC120	2017 January	H6	CCU2	RT014	26	11	5	4	15	16	C680 → T/ MH753340	+/-/-	WT/ MH769645	Asp426 → Asn/ MH796027

PC121	2017 February	H3	Infectious disease	RT001	33	5	16	10	14	17	-	+/-/-	Thr82 → Val/ MH769618	WT/ MH796061
PC122	2017 February	H6	CCU2	RT266	30	9	2	15	17	55	-	+/-/-	WT/ MH769646	WT/ MH796062
PC122b	2017 March	H6	CCU2	RT266	22	9	2	8	17	56	-	+/+/-	WT/ MH769647	WT/ MH796063
PC123	2017 February	H3	Surgery	RT072	23	5	16	8	15	18	C680 → T/ MH753341	+/+/-	Thr82 → Val/ MH769619	WT/ MH796064
PC124	2017 February	H4	Respiratory diseases	RT001	46	5	16	8	14	19	-	+/+/-	Thr82 → Val/ MH769620	WT/ MH796065
PC125	2017 February	H9	ICU	RT001	28	5	14	8	16	20	C680 → T/ MH753342	+/-/-	Thr82 → Val/ MH769621	WT/ MH796066
PC126	2017 February	H9	ICU	RT072	35	5	14	8	15	21	C680 → T/ MH753343	+/-/-	Thr82 → Val/ MH769622	WT/ MH796067
PC127	2017 April	H3	ICU	RT131	38	5	1	10	14	22	C680 → T/ MH753344	+/-/-	WT/ MH769648	WT/ MH796068
PC128	2017 April	H9	Infectious disease	RT668	22	9	1	9	11	23	C680 → T/ MH753345	+/-/-	WT/ MH769649	WT/ MH796069
PC129	2017 April	H4	ICU	RT029	20	12	2	6	8	24	C680 → T/ MH753346	+/-/-	WT/ MH769650	WT/ MH796070

PC130	2017 May	H3	ICU	RT005	25	7	5	11	18	25	C680 → T/ MH753347	+/-/-	WT/ MH769651	WT/ MH796071
PC131	2017 May	H3	Liver transplantation	RT014	25	9	4	7	13	26	-	+/-/-	WT/ MH769652	WT/ MH796072
PC132	2017 May	H9	Infectious disease	RT001	27	5	14	8	16	27	WT/ MH753348	+/-/-	Thr82 → Val/ MH769623	WT/ MH796073
PC133	2017 June	H9	Surgery 2	RT001	29	5	14	8	16	28	C680 → T/ MH753349	+/+/-	Thr82 → Val/ MH769624	WT/ MH796074
PC134	2017 June	H9	Internal (ward 9)	RT369	26	34	2	8	13	54	-	+/-/-	WT/ MH769625	Ser366 → Ala, Asp426 → Val/ MH796028
PC136	2017 June	H3	ICU	RT001	25	5	14	8	13	29	-	+/-/-	WT/ MH769626	WT/ MH796075
PC139	2017 July	H6	CCU4	RT001	22	6	14	8	14	53	-	+/-/-	Thr82 → Val/ MH769627	WT/ MH796076
PC140	2017 July	H3	Endocrine	RT001	30	6	16	9	14	30	C680 → T/ MH753350	+/-/-	WT/ MH769628	WT/ MH796077
PC141	2017 July	H3	Medical 2	unidentified RT	32	19	3	5	14	50	-	+/+/-	WT/ MH769629	WT/ MH796078

[a] Not recorded (NR)
[b] Negative result in PCR amplification (-)
[c] Wild type (WT)
